# Epidemiological Research in Physical Activity and Sedentary Behaviors

**DOI:** 10.1155/2018/3527439

**Published:** 2018-11-28

**Authors:** Wonwoo Byun, Jung-Min Lee, Yang Bai, Youngdeok Kim

**Affiliations:** ^1^Department of Health, Kinesiology, and Recreation, University of Utah, Salt Lake City, UT, USA; ^2^School of Physical Education, Kyung Hee University, Yongin, Republic of Korea; ^3^Department of Rehabilitation and Movement Science, University of Vermont, Burlington, VT, USA; ^4^Department of Kinesiology and Sport Management, Texas Tech University, Lubbock, TX, USA

Accumulating data from population-based epidemiological studies is needed to demonstrate positive health outcomes associated with regular physical activity across lifespans. It becomes clearer that physical activity plays a critical role in preventing a number of chronic diseases, such as cardiovascular disease [1], type 2 diabetes [2], obesity [3], osteoporosis [4], and cancer [5], as well as premature mortality [6, 7]. To date, unfortunately, only a small proportion of the population in developed and/or developing countries is physically active enough to gain the associated health benefits. Thus it places physical inactivity (i.e., lack of physical activity) as one of the major public health concerns worldwide.

Further, more recently, there is growing evidence concerning sedentary behavior, independent of physical inactivity, as an emerging health risk behavior in contemporary societies. Recent studies showed that not only the total volume of time spent being sedentary, but also the manner in which sedentary time is accumulated are associated with health outcomes [6, 8]. However, available data are limited and sometimes inconsistent, leaving a gap in understanding the role of sedentary behavior on health in various population groups. As part of continuous efforts to extend our understanding in two of the most influencing lifestyle factors on human health, physical activity, and sedentary behaviors, this special issue focuses on a broad range of topics in epidemiological research on physical activity and sedentary behavior within a behavioral epidemiology framework.

We invited investigators to submit original research articles as well as review articles addressing recent advances in epidemiological studies defining physical activity and/or sedentary behavior as either an exposure or an outcome variable. More specifically, this special issue is dedicated (1) to understanding how physical activity and/or sedentary behavior independently and/or jointly influence the risk of developing adverse health outcomes and longevity in various population groups, (2) to exploring the factors at various levels (e.g., individual, environmental) influencing physical activity and sedentary behavior, (3) to improving physical activity and sedentary behavior assessments in epidemiological research; and (4) to exploring the evidence-based intervention strategies to modify the behaviors at various population groups.

Given the significant burden and the increasing prevalence of chronic disease, the social significance of physical activity and sedentary behavior have never been greater. This special issue has strived to include, in a comprehensive manner, novel research employing methodologies in epidemiology studies such as randomized controlled trials, community-based interventions, observational study, and systematic reviews. With the recognition of difficulties in translating scientific knowledge generated from research into public health practice, we believe that the knowledge provided by this special issue would further contribute to generating high-quality evidence on the health benefits of physical activity and promoting physical activity in practical settings.
